# Uso de enxerto como reforço no reparo do manguito rotador: Um protocolo de revisão sistemática

**DOI:** 10.1055/s-0045-1814405

**Published:** 2025-12-30

**Authors:** Victor Peyneau Poncio, Bianca Carolina Bankhardt da Silva, Fabio Teruo Matsunaga, Nicola Archetti Netto, João Carlos Belloti, Marcel Jun Sugawara Tamaoki

**Affiliations:** 1 Departamento de Ortopedia e Traumatologia, Escola Paulista de Medicina, Universidade Federal de São Paulo, São Paulo, SP, Brasil

**Keywords:** alicerces teciduais, aloenxerto, autoenxertos, lesões do manguito rotador, manguito rotador, xenoenxertos, allografts, autografts, heterografts, rotator cuff, rotator cuff injuries, tissue scaffolds

## Abstract

**Objetivo:**

Avaliar a eficácia do uso de enxerto como reforço no reparo do manguito rotador comparado ao não uso, e a eficácia de diferentes tipos de enxerto, considerando a cicatrização e a função do ombro como desfechos primários, por meio de uma revisão sistemática e metanálise de ensaios clínicos randomizados (ECRs).

**Métodos:**

Realizaremos uma revisão sistemática de ECRs conforme as diretrizes do *Cochrane Handbook for Systematic Reviews of Interventions* e da declaração de Preferred Reporting Items for Systematic reviews and Meta-Analyses for Protocols (PRISMA-P). Serão incluídos apenas ECRs com participantes adultos que comparem o reparo com enxerto como reforço e o reparo-padrão, ou que comparem diferentes tipos de enxertos. Será realizada uma busca eletrônica nas bases MEDLINE (PubMed), Cochrane Central Register of Controlled Trials (CENTRAL), Embase e SciELO, além de literatura cinzenta. O gerenciamento e extração de dados serão conduzidos com formulário padronizado que avaliará as características do estudo, dos participantes e da intervenção, além dos resultados e dos domínios metodológicos. Os desfechos primários serão a cicatrização e a avaliação funcional do ombro, e os secundários, dor, amplitude de movimento, eventos adversos, complicações, qualidade de vida e a relação custo-eficácia. O risco de viés será avaliado por meio do Risk of Bias, versão 2.0 (RoB 2.0), e a certeza da evidência, por meio da abordagem Grading of Recommendations Assessment, Development, and Evaluation (GRADE). Quando aplicável, serão realizadas, análises descritivas, de subgrupos e de sensibilidade.

**Conclusão:**

Esta revisão espera consolidar evidências sobre a eficácia do uso de enxerto como reforço no reparo do manguito rotador. Este estudo foi inscrito no International Prospective Register of Systematic Reviews (PROSPERO) sob o número 1106892.

## Abstract (en)

**Objective:**

To evaluate the effectiveness of using a graft for reinforcement in rotator cuff repair compared to no use, as well as the effectiveness of different types of grafts, considering healing and shoulder function as primary outcomes, through a systematic review and meta-analysis of randomized controlled trials (RCTs).

**Methods:**

We will conduct a systematic review of RCTs following the guidelines of the *Cochrane Handbook for Systematic Reviews of Interventions* and of the Preferred Reporting Items for Systematic reviews and Meta-Analyses for Protocols (PRISMA-P) statement. Only RCTs with adult participants comparing repair with a reinforcing graft versus the standard repair, or comparing different types of grafts, will be included. An electronic search will be conducted in the MEDLINE (PubMed), Cochrane Central Register of Controlled Trials (CENTRAL), Embase, and SciELO databases, as well as gray literature. Data management and extraction will be performed using a standardized form that will assess the characteristics of the studies, participants, and intervention, as well as the outcomes and methodological domains. The primary outcomes will be healing and shoulder functional assessment, and the secondary outcomes, pain, range of motion, adverse events, complications, quality of life, and cost-effectiveness. Risk of bias will be assessed with the Risk of Bias, version 2.0 (RoB 2.0), and the certainty of evidence, through the Grading of Recommendations Assessment, Development, and Evaluation (GRADE) approach. Descriptive, subgroup, and sensitivity analyses will be performed when applicable.

**Conclusion:**

The review is expected to consolidate evidence on the effectiveness of using a graft for reinforcement in rotator cuff repair. It has been registered in the International Prospective Register of Systematic Reviews (PROSPERO) under number 1106892.

## Introdução

Lesões do manguito rotador representam um desafio crescente na cirurgia do ombro, com prevalência crescente devido ao envelhecimento demográfico. 1 Tais lesões são a principal patologia do ombro, e as cirurgias para tratá-las são as mais realizadas no contexto epidemiológico de patologias do ombro. 2 A taxa de falha do reparo, apesar do constante avanço técnico e aprimoramento científico, mostra-se significativa, e varia entre 30 e 40% nas cirurgias primárias, com estudos 3 4 que evidenciam valores preditivos de 80 a 90% de não cicatrização, a depender de fatores importantes, como a cicatrização, o tamanho da lesão e o grau de atrofia da musculatura do manguito rotador. Esse panorama impacta diretamente a qualidade de vida dos pacientes e a necessidade de reintervenções, o que eleva os custos da saúde. No contexto da saúde dos Estados Unidos, 5 os custos indiretos da falha do reparo do manguito rotador são estimados em centenas de milhares de dólares.

Diferentes estratégias têm sido exploradas para minimizar as falhas, incluindo o uso de técnicas como reparo em dupla fileira, reconstrução capsular superior e transferências tendíneas. 6 7 Entretanto, a colocação de materiais para reforço, denominados *enxertos* ou *patches* , tem ganhado destaque. Esses materiais podem ser biológicos (provenientes de humanos e animais) ou sintéticos, e atuam de maneira a melhorar o suporte cicatricial e o reforço mecânico, com o objetivo de promover melhor cicatrização e resistência do reparo. 8

Pesquisas recentes 9 10 sugerem redução de até 30% nas taxas de ruptura e melhora nos escores funcionais com o uso de reforço. Uma pesquisa com cirurgiões do Reino Unido 11 revelou que 70% daqueles que utilizam reforço, o fazem em lesões grandes ou extensas, mas ainda há divergência quanto ao melhor tipo de enxerto e a indicação ideal.

Apesar de evidências em diferentes estudos, há falta de revisões sistemáticas recentes baseadas em ensaios clínicos randomizados (ECRs), o que justifica a condução de uma revisão sistemática com metanálise baseada exclusivamente neles. Foram encontradas somente 3 revisões prévias na literatura, mas todas com mais de 5 anos de publicação, e que avaliavam ensaios com nível de evidência baixo, como os não randomizados e as séries de casos, assim como estudos retrospectivos. A estratégia geral será desenvolvida em torno dos conceitos-chave da estratégia de População, Intervenção, Comparação, Desfecho (“Outcome”, em inglês), e Delineamento Experimental (“Study Design”, em inglês) PICOS: população – pacientes com lesões do manguito rotador; intervenção – reforço com enxerto; comparação – reparo sem reforço (controle) ou com outro tipo de reforço com enxerto; desfecho – cicatrização tendínea e desfechos funcionais; e delineamento experimental – revisão sistemática.

Portanto, o objetivo deste estudo é comparar a eficácia do uso ou não do enxerto no reforço, além da eficácia dos diferentes tipos de enxerto, no reparo do manguito rotador, considerando a cicatrização e a função do ombro, por meio de uma revisão sistemática e metanálise de ECRs.

### Tipo de Estudo

Este estudo é uma revisão sistemática de ECRs que avaliam a eficácia da cicatrização tendínea e os desfechos funcionais em adultos com lesões do manguito rotador submetidos a cirurgia de reparo com uso de reforço, avaliando qualquer tipo de enxerto (biológico ou sintético). O estudo seguirá as recomendações propostas pelo *Cochrane Handbook for Systematic Reviews of Interventions* e pela declaração de Preferred Reporting Items for Systematic Reviews and Meta-Analyses for Protocols (PRISMA-P) ( Anexo 1 ). 12 13

**Anexo 1:** Lista de verificação da declaração PRISMA-P 2015

Tópico/Sessão	N° do item	Item da lista	N° da página
**INFORMAÇÕES ADMINISTRATIVAS**			
Título			
Identificação	1a	Identificar o relatório como um protocolo de uma revisão sistemática	1
Atualização	1b	Se o protocolo for para uma atualização de uma revisão sistemática anterior, identificar como tal	–
Registro	2	Se registrado, fornecer o nome do registro (como PROSPERO) e o número de registro no resumo	1
Autores			
Contato	3a	Fornecer o nome, afiliação institucional e endereço de e-mail de todos os autores do protocolo; fornecer o endereço postal físico do autor correspondente	Folha de rosto
Contribuições	3b	Descrever as contribuições dos autores do protocolo e identificar o fiador da revisão	Folha de rosto
Emendas	4	Se o protocolo representar uma emenda de um protocolo previamente concluído ou publicado, identificá-lo como tal e listae as alterações; caso contrário, indicar um plano para documentar emendas importantes para o protocolo	–
Apoio			
Fontes	5a	Indicar fontes de apoio financeiro ou outro para a revisão	Folha de rosto
Patrocinador	5b	Fornecer o nome do financiador e/ou patrocinador da revisão	Folha de rosto
Função do patrocinador ou financiador	5c	Descrever as funções do(s) financiador(es), patrocinador(es) e/ou instituição(ões), se houver, no desenvolvimento do protocolo	Folha de rosto
**INTRODUÇÃO**			
Justificativa	6	Descrever a justificativa para a revisão no contexto do que já é conhecido	4
Objetivos	7	Fornecer uma declaração explícita da(s) questão(ões) que a revisão abordará com referência ao PICOS	4
**MATERIAIS E MÉTODOS**			
Critérios de elegibilidade	8	Especificar as características do estudo (como PICOS, cenário, período) e as características do relatório (como anos considerados, idioma, *status* da publicação) a serem usadas como critérios de elegibilidade para a revisão	5
Fontes de informação	9	Descrever todas as fontes de informação pretendidas (como bancos de dados eletrônicos, contato com autores do estudo, registros de ensaios ou outras fontes de literatura cinzenta) com datas planejadas de cobertura	7
Estratégia de busca	10	Apresentar rascunho da estratégia de busca a ser usada para pelo menos um banco de dados eletrônico, incluindo limites planejados, de modo que possa ser repetida	7
Registros de estudo			
Gerenciamento de dados	11a	Descrever o(s) mecanismo(s) que serão usados para gerenciar registros e dados durante a revisão	8
Processo de seleção	11b	Indicar o processo que será usado para selecionar estudos (como dois revisores independentes) em cada fase da revisão (ou seja, triagem, elegibilidade e inclusão em metanálise)	8
Processo de coleta de dados	11c	Descrever o método planejado para extrair dados de relatórios (como formulários-piloto, feitos de forma independente, em duplicata), e quaisquer processos para obter e confirmar dados de pesquisadores	8
Itens de dados	12	Listar e definir todas as variáveis para as quais os dados serão buscados (como itens PICOS, fontes de financiamento), e quaisquer suposições e simplificações de dados pré-planejadas	8,9
Resultados e priorização	13	Listar e definir todos os resultados para os quais os dados serão buscados, incluindo a priorização dos resultados principais e adicionais, com justificativa	9
Risco de viés em estudos individuais	14	Descrever métodos previstos para avaliar o risco de viés de estudos individuais, incluindo se isso será feito no nível do resultado ou do estudo, ou em ambos; e declarar como essas informações serão usadas na síntese de dados	10
Síntese de dados	15a	Descrever os critérios sob os quais os dados do estudo serão sintetizados quantitativamente	12
	15b	Se os dados forem apropriados para síntese quantitativa, descrever as medidas de resumo planejadas, métodos de tratamento de dados e métodos de combinação de dados de estudos, incluindo qualquer exploração planejada de consistência (como I ^2^ , Tau de Kendall)	9
	15c	Descrever quaisquer análises adicionais propostas (como análises de sensibilidade ou de subgrupo, metarregressão)	11
	15d	Se a síntese quantitativa não for apropriada, descrever o tipo de resumo planejado	10
Metaviés(es)	16	Especificar qualquer avaliação planejada de metaviés(es) (como viés de publicação entre estudos, relatórios seletivos dentro de estudos)	10
Confiança na evidência cumulativa	17	Descrever como a força do conjunto de evidências será avaliada (como GRADE)	11

**Abreviaturas:** GRADE, Grading of Recommendations Assessment, Development, and Evaluation; PICOS, População, Intervenção, Comparação, “Outcome” (“Desfecho”) e “Study Design” (“Delineamento Experimental”); PRISMA-P, Preferred Reporting Items for Systematic reviews and Meta-Analyses for Protocols; PROSPERO, International Prospective Register of Systematic Reviews.

### Ética e Registros

O estudo foi inscrito no International Prospective Register of Systematic Reviews (PROSPERO) sob o número 1106892 e com o título “Patch Augmentation in Rotator Cuff Repair: A Systematic Review and Meta-analysis of Randomized Controlled Trials.”

### Critérios de Inclusão e Exclusão

Os artigos elegíveis são ECRs ou ensaios quase-randomizados, com participantes adultos (maiores de 18 anos) submetidos ao tratamento de reparo total do manguito rotador, que comparam o uso de enxerto como reforço associado ao reparo isolado ou comparado a outro enxerto.

Os seguintes critérios de exclusão serão adotados: estudos clínicos não randomizados, não comparativos, em cadáveres, de coorte, observacionais, relatos de caso, estudos de caso-controle, e aqueles realizados em animais. Estudos com procedimentos adicionais sem reparo do manguito rotador associado ou uso de enxerto com outro fim (como para a reconstrução da cápsula superior, por exemplo) também serão excluídos.

### Desfechos Primários

Os desfechos primários serão a cicatrização do manguito rotador e a função do ombro.

A cicatrização tendínea será avaliada por métodos de imagem, ressonância magnética (RM) ou ultrassonografia (USG). A avaliação da integridade estrutural será feita de maneira dicotômica (presença ou ausência de rerruptura) e por critérios de classificação padronizados e validados na literatura, como o proposto por Sugaya et al., 14 classificação amplamente empregada para descrever a integridade estrutural pós-operatória, 14 além da avaliação do número absoluto de rerrupturas.

A função do ombro será avaliada a partir do acompanhamento pós-operatório, fazendo uso de escores validados, como o Constant-Murley Score (CMS), 15 o American Shoulder and Elbow Surgeons Society Standardized Shoulder Assessment Score (ASES), 16 o Penn Shoulder Score (PSS), 17 e o questionário de Disabilities of the Arm, Shoulder and Hand (DASH). 18 Caso a função do ombro seja avaliada usando outros escores validados, estes também serão considerados, e, se aplicável, suas medidas serão convertidas para uma métrica comum, conforme apropriado.

Para avaliar a implicação clínica dos resultados obtidos, será utilizado o conceito de diferença mínima clinicamente importante (DMCI), o qual consiste em um modelo estatístico que procura definir a menor mudança em um escore ou escala que seja identificada pelo paciente como uma alteração significativa no seu estado de saúde.

### Desfechos Secundários

Como desfechos secundários, também serão avaliados a dor (por meio de escalas validadas), a amplitude de movimento, e escores de qualidade de vida relacionados, tal qual o 36-Item Short Form Survey Instrument (SF-36). Adicionalmente, o estudo investigará e registrará eventos adversos, complicações pós-operatórias e situações de má evolução, incluindo, mas não se limitando a, infecções, rigidez persistente e necessidade de reintervenção cirúrgica.

Todos os desfechos de serão avaliados seguindo a métrica de período: curto prazo (até 6 meses), médio prazo (6 meses a 2 anos), e longo prazo (mais de 2 anos).

### Métodos e Estratégias de Busca

A busca eletrônica será feita nas bases de dados MEDLINE (via PubMed), Cochrane Central Register of Controlled Trials (CENTRAL), Embase, SciELO. Adicionalmente, será avaliada a literatura cinzenta, como Google Scholar e as listas de referências dos estudos incluídos, além dos anais dos principais congressos sobre o assunto, para buscar estudos adicionais relevantes não capturados na busca eletrônica primária. Não serão impostas restrições de idioma ou data de publicação.

As estratégias de busca serão desenvolvidas para maximizar a sensibilidade e a especificidade da busca. Para cada base de dados, será desenvolvida uma pesquisa combinando termos controlados (quando aplicável, como medical subject headings [MeSH] na PubMed/MEDLINE e Emtree na Embase) e termos de texto livre (palavras-chave e sinônimos), utilizando operadores booleanos (AND e OR). A estratégia geral será desenvolvida em torno dos conceitos-chave da estratégia PICOS ( Anexo 2 ).

**Anexo 2:** Estratégia de busca

Base de dados	Termos de busca	Filtros recomendados
MEDLINE (via PubMed)	( *Rotator Cuff* [Mesh] OR *Rotator Cuff Injuries* [Mesh] OR *rotator* cuff tear* OR rotator cuff injur* OR massive rotator cuff tear* OR large rotator cuff tear* ) AND ( *Surgical Procedures, Operative* [Mesh] OR *rotator cuff repair* OR *arthroscopic repair* ) AND ( *Biocompatible Materials* [Mesh] OR *Tissue Scaffolds* [Mesh] OR *patch* OR *graft* OR *augmentation* OR *scaffold* OR *biologic augmentation* OR *synthetic patch* OR *acellular dermal matrix* ) AND ( *Randomized Controlled Trial* [Publication Type] OR *randomized controlled trial* OR *RCT* )	Humanos; adultos; ensaios clínicos randomizados
Cochrane Central Register of Controlled Trials (CENTRAL)	( *rotator cuff* OR *rotator cuff tear* OR *rotator cuff injuries* ) AND ( *augmentation* OR *patch* OR *graft* OR *scaffold* OR *acellular dermal matrix* OR *biologic augmentation* OR *synthetic patch* ) AND ( *rotator cuff repair* OR *surgery* OR *arthroscopic repair* )	‘ *Ensaios* > *randomizados* ’
Emabse	( *rotator cuff injury/exp* OR *rotator cuff tear* OR *rotator cuff repair* OR *massive rotator cuff tear* ) AND ( *augmentation/exp* OR *tissue scaffold/exp* OR *patch* OR *graft* OR *acellular dermal matrix* OR *synthetic patch* OR *biologic augmentation* ) AND ( *randomized controlled trial/exp* OR *randomized controlled trial* )	Tipo de estudo: ensaio clínico randomizado; somente humanos; idade: adulto
SciELO	( *manguito rotador* OR *lesão do manguito rotador* OR *rotator cuff* ) AND ( *enxerto* OR patch" OR *aumento* OR *augmentação* OR *scaffold* OR *matriz dérmica acelular* ) AND ( *ensaio clínico randomizado* OR *estudo clínico randomizado* OR *randomized controlled trial* )	Tipo de artigo: ensaio clínico

### Coleta, Extração e Análise de Dados

Dois autores selecionarão independentemente os estudos elegíveis para esta revisão sistemática por meio do título e do resumo usando os seguintes critérios: 1) ECRs; 2) presença de lesão total do manguito rotador; 3) reparo cirúrgico com uso de enxerto (biológico, animal ou humano, e sintético) como reforço; e 4) grupo de controle comparativo, seja sem uso de reforço ou com uso de diferentes enxertos. A revisão se dará em duas fases, e analisará primariamente o título e o resumo e, após a inclusão dos estudos, será feita a leitura do texto completo. Os estudos selecionados serão revisados integralmente para determinar sua elegibilidade, e qualquer discordância será resolvida por meio de discussão e, quando necessário, será julgada por um terceiro autor na tentativa de resolver um possível conflito. Dois revisores farão a extração de dados, os quais serão dispostos em um formulário personalizado apropriado no programa Excel (Microsoft Corp.), versão 16.34, com base: 1) nas características dos estudos, incluindo autores, ano de publicação, tamanho da amostra (total e por grupo), perda de seguimento, características sociodemográficas dos participantes (idade média, sexo, comorbidades, entre outras), número de pacientes recrutados, critérios de inclusão e exclusão, e tempo de acompanhamento; 2) nas características da intervenção, como tipo de cirurgia, uso de qual enxerto para reforço; 3) nos resultados durante o acompanhamento pós-operatório relativos à função (escores funcionais), assim como dor, amplitude de movimento, cicatrização tendínea e complicações; e 4) domínios metodológicos e viés.

### Tratamento dos Dados

Os dados dicotômicos resultantes serão analisados por meio de razão de chances (RC) e IC95%. Dados sobre resultados contínuos serão expressos como uma diferença média (DM) ou diferença média padronizada (DMP), com IC95%. Os resultados serão agrupados com a DM se dois ou mais ensaios revelarem resultados do mesmo instrumento validado de avaliação (com as mesmas unidades de medida). Se os estudos primários medirem os mesmos resultados, como a função do ombro por meio de diferentes instrumentos (bem como diferentes unidades de medida), a DM será transformada em DMP, para permitir a avaliação dos resultados, independentemente das unidades de medida originais.

A heterogeneidade estatística será avaliada usando o teste Q de Cochran e o índice I ^2^ . O teste Q de Cochran será utilizado para verificar a significância estatística da heterogeneidade, com um valor de *p* < 0,10 sendo considerado indicativo de heterogeneidade significativa, devido à sua baixa potência em metanálises com poucos estudos. Já o índice I ^2^ será utilizado para quantificar a magnitude da heterogeneidade entre os estudos, ao expressar a porcentagem da variação total dos efeitos observados que se deve à heterogeneidade verdadeira, não ao acaso. A interpretação do I ^2^ será conforme as seguintes diretrizes: 0 a 40% (pode não ser importante); 30 a 60% (heterogeneidade moderada); 50 a 90% (heterogeneidade substancial); e 75 a 100% (heterogeneidade considerável). Um modelo de efeitos aleatórios será utilizado para agrupar os resultados, devido à provável heterogeneidade clínica e metodológica entre os estudos incluídos, mesmo quando o teste de heterogeneidade não for estatisticamente significativo. O programa Review Manager (RevMan, The Cochrane Collaboration), versão 5.3, será usado para as análises estatísticas.

Quando houver disponibilidade de dados quantitativos para desfechos de interesse, com métricas padronizáveis, e a heterogeneidade entre os estudos for considerada aceitável, será realizada a metanálise. Em caso de heterogeneidade significativa, serão exploradas análises de subgrupos e/ou metarregressão, se o número de estudos permitir. Quando aplicável e com número suficiente de estudos, serão consideradas metanálises pareadas e em rede para cada desfecho.

### Avaliação do Risco de Viés

Dois autores avaliarão independentemente aspectos da confiabilidade metodológica dos estudos utilizando a ferramenta Risk of Bias 2.0 (RoB 2, The Cochrane Collaboration), 19 que é para ECRs, considerando os quatro principais domínios abordados (viés por desvios da intervenção planejada, viés devido a dados de desfecho ausentes, viés na mensuração dos desfechos, e viés na seleção dos resultados relatados). Divergências entre os revisores serão resolvidas por consenso ou por um terceiro revisor.

### Análise de Sensibilidade

A análise de sensibilidade consistirá na exclusão progressiva de estudos com características metodológicas heterogêneas ou fatores de confusão que possam comprometer a validade dos resultados. Estudos excluídos na análise de sensibilidade não serão removidos da análise principal, mas serão utilizados para avaliar o impacto potencial sobre a estimativa global de efeito. Em análises quantitativas com número suficiente de estudos, poderá ser utilizado gráfico de funil, associado aos testes de Egger ou de Begg para investigar o viés de publicação, observando-se a simetria na distribuição dos efeitos.

### Qualidade da Evidência

Adicionalmente, a qualidade da evidência para cada desfecho crítico será avaliada segundo a abordagem Grading of Recommendations Assessment, Development and Evaluation (GRADE). 20

Os seguintes critérios serão considerados para a graduação da evidência no aspecto de qualidade: 1) viés por desvios das intervenções planejadas; 2) viés devido a dados de desfecho ausentes; 3) viés na mensuração dos desfechos; 4) viés na seleção dos resultados relatados; 5) viés devido ao processo de randomização; e 6) viés devido à ocultação da alocação e cegamento.

A evidência será classificada como de alta, moderada, baixa ou muito baixa qualidade. Adicionalmente, a qualidade da evidência poderá ser aumentada com base em três fatores: magnitude elevada do efeito, relação dose-resposta e ausência de vieses que expliquem o efeito observado.

### Dados Ausentes

Uma análise de intenção de tratar será realizada para incluir todos os participantes randomizados de qualquer intervenção. Os autores dos ensaios selecionados serão contatados em caso de informações insuficientes. Uma análise será realizada independentemente da perda de dados, de acordo com o pior e o melhor cenários.

### Análise de Subgrupos

Os subgrupos serão analisados para explorar a diferença nos desfechos de cada enxerto: comparação entre o uso e o não uso de enxerto em associação ao reparo do manguito rotador; comparação entre o uso de enxertos biológicos e sintéticos; além de outras comparações relevantes para a compreensão dos desfechos entre as técnicas disponíveis. Além disso, será avaliado o resultado para os subgrupos conforme o tamanho da lesão e o número de tendões acometidos, caso seja possível.

### Síntese de Dados

A síntese de dados incluirá uma descrição das características dos estudos incluídos (como população, intervenção, comparação, desfechos e risco de viés, por exemplo), para facilitar a identificação de estudos passíveis de meta-análise. Para o relatório estruturado dos efeitos, o modelo de efeito aleatório e um IC95% serão adotados. Serão incluídas as considerações da direção do efeito, da magnitude do efeito, da certeza da evidência, bem como das intervenções testadas e das populações nas quais foram testadas. O modelo de variáveis será usado quando houver diversidade nas características clínicas ou metodológicas.

## Discussão

Lesões do manguito rotador são uma das principais patologias do ombro; nesse contexto, o reparo apresenta-se como uma das cirurgias mais realizadas. As altas taxas de falha do reparo, seja por rerrotura ou não cicatrização tendínea, são um desafio que persiste. A partir disso, tem-se um impacto importante na qualidade de vida do paciente e na necessidade de reintervenção, sendo essencial avaliar a eficácia de diferentes estratégias de tratamento. O uso de enxerto é uma técnica explorada que atua como reforço mecânico e biológico para o reparo.

Uma revisão sistemática e metanálise de ECRs representa alto nível de evidência científica para avaliar a eficácia de tratamentos. Como desfechos primários, utilizaremos medidas objetivas de cicatrização tendínea via RM ou USG, e melhora funcional usando escores validados na literatura. Essa combinação fornece um panorama do sucesso clínico e estrutural do procedimento. A avaliação em períodos de curto, médio e longo prazos fornece uma evidência sobre a longevidade dos resultados. Adicionalmente, investigaremos desfechos secundários, incluindo dor, amplitude de movimento, complicações e eventos adversos. O uso de análises de subgrupos e a avaliação de heterogeneidade permite observar diferenças nos desfechos entre os diferentes tipos de enxertos e tamanhos de lesão, uma vez que a magnitude do benefício pode ser influenciada pelo material do enxerto, pela técnica empregada e pelas características do paciente, o que reforça a necessidade de recomendações.

Considerando que a literatura atual aborda diversas opções de tratamento para lesões do manguito rotador, ainda faltam evidências que apoiem a prática clínica com elevado nível de evidência científica. Portanto, esta revisão sistemática irá consolidar a evidência existente e esclarecer o papel dessa técnica no reparo do manguito rotador.
